# An unexpected twist to the activation of IKKβ: TAK1 primes IKKβ for activation by autophosphorylation

**DOI:** 10.1042/BJ20140444

**Published:** 2014-07-10

**Authors:** Jiazhen Zhang, Kristopher Clark, Toby Lawrence, Mark W. Peggie, Philip Cohen

**Affiliations:** *MRC Protein Phosphorylation and Ubiquitylation Unit, College of Life Sciences, University of Dundee, Dundee DD1 5EH, Scotland, U.K.; †Centre d’Immunologie de Marseille-Luminy, Parc Scientifique & Technologique de Luminy, Case 906, 13288 Marseille cedex 09, France

**Keywords:** inhibitor of nuclear factor κB kinase (IKK), interleukin-1 (IL-1), linear ubiquitin chain assembly complex (LUBAC), nuclear factor κB (NF-κB), transforming growth factor β-activated kinase-1 (TAK1), BMDM, bone-marrow-derived macrophage, E, embryonic day, HA, haemaglutinnin, HEK, human embryonic kidney, HOIP, HOIL1 [haem-oxidized IRP2 (iron regulatory protein 2) ubiquitin ligase 1]-interacting protein, IκB, inhibitor of NF-κB, IKK, IκB kinase, IL-1, interleukin-1, JNK, c-Jun N-terminal kinase, LPS, lipopolysaccharide, LUBAC, linear ubiquitin chain assembly complex, MAPK, mitogen-activated protein kinase, M-CSF, macrophage colony-stimulating factor, MEF, mouse embryonic fibroblast, MKK, MAPK kinase, NEMO, NF-κB essential modulator, NF-κB, nuclear factor κB, PP1γ, protein phosphatase 1γ, TAB, TAK1-binding protein, TAK1, transforming growth factor β-activated kinase-1, TLR, Toll-like receptor, TNF, tumour necrosis factor, TRAF, TNF receptor-associated factor

## Abstract

IKKβ {IκB [inhibitor of NF-κB (nuclear factor κB)] kinase β} is required to activate the transcription factor NF-κB, but how IKKβ itself is activated *in vivo* is still unclear. It was found to require phosphorylation by one or more ‘upstream’ protein kinases in some reports, but by autophosphorylation in others. In the present study, we resolve this contro-versy by demonstrating that the activation of IKKβ induced by IL-1 (interleukin-1) or TNF (tumour necrosis factor) in embryonic fibroblasts, or by ligands that activate Toll-like receptors in macrophages, requires two distinct phosphorylation events: first, the TAK1 [TGFβ (transforming growth factor β)-activated kinase-1]-catalysed phosphorylation of Ser^177^ and, secondly, the IKKβ-catalysed autophosphorylation of Ser^181^. The phosphorylation of Ser^177^ by TAK1 is a priming event required for the subsequent autophosphorylation of Ser^181^, which enables IKKβ to phosphorylate exogenous substrates. We also provide genetic evidence which indicates that the IL-1-stimulated, LUBAC (linear ubiquitin chain assembly complex)-catalysed formation of linear ubiquitin chains and their interaction with the NEMO (NF-κB essential modulator) component of the canonical IKK complex permits the TAK1-catalysed priming phosphorylation of IKKβ at Ser^177^ and IKKα at Ser^176^. These findings may be of general significance for the activation of other protein kinases.

## Abstract 2

We have discovered how a key enzyme that controls the immune system is switched on during infection by bacteria and viruses. Known by the acronym IKKβ, it triggers the production of many proteins that are needed to combat these pathogens.

## INTRODUCTION

The canonical IKK {IκB [inhibitor of NF-κB (nuclear factor κB)] kinase β} complex, consisting of the protein kinases IKKα and IKKβ (also called IKK1 and IKK2) and a regulatory component called NEMO (NF-κB essential modifier) [1,2], is one of the most studied of all protein kinases. It has featured in over 10000 papers since its discovery in 1998 due to its essential role in activating NF-κB, a ‘master’ transcription factor that regulates many physiological processes, including innate immunity and the cellular response to DNA damage [3–5]. Nevertheless, despite the vast number of publications that have focused on this protein kinase, its mechanism of activation is still controversial.

The activation of IKKα and IKKβ requires phosphorylation of the ‘activation loops’ of these protein kinases at Ser^176^ and Ser^180^ (IKKα) or Ser^177^ and Ser^181^ (IKKβ) [4]. The IKKs respond to many physiological stimuli, but are activated most powerfully by inflammatory stimuli, such as TLR (Toll-like receptor) agonists and the pro-inflammatory cytokines IL-1 (interleukin-1) and TNF (tumour necrosis factor). Genetic evidence indicates that the expression and activity of the TAK1 [TGFβ (transforming growth factor β)-activated kinase-1; also called MAP3K7 (mitogen-activated protein kinase kinase kinase 7)] is needed for the activation of the canonical IKK complex by IL-1 and TNF in MEFs (mouse embryonic fibroblasts). These agonists fail to activate the IKKs in MEFs that do not express the TAK1 catalytic subunit [6] or that express a truncated inactive form of TAK1 [7]. IL-1 and TNF trigger TAK1 activation within minutes, a speed compatible with a role in initiating the activation of the IKKs [8]. TAK1 is also reported to phosphorylate and activate the canonical IKKs *in vitro* [9], activation being prevented by pharmacological inhibitors of TAK1 [8,10,11]. Similar lines of evidence indicate an essential role for TAK1 in activating the MKKs [MAPK (mitogen-activated protein kinase) kinases] that switch on the MAPK family members JNK1 (c-Jun N-terminal kinase 1) and JNK2 and p38 MAPKs in MEFs [8–11].

On the other hand, the canonical IKKs have been shown to be capable of phosphorylating and activating themselves *in vitro* (reviewed in [4]). For example, Met^1^-linked (also called linear) ubiquitin oligomers [12] and other types of ubiquitin oligomers [13] have been reported to induce the activation of the canonical IKK complex *in vitro*, apparently in the absence of any ‘upstream’ activating protein kinase. These observations raise the alternative possibility that the role of TAK1 *in vivo* might be to stimulate the formation of these polyubiquitin chains, rather than to phosphorylate the canonical IKK complex directly. In addition, X-ray crystallographic analysis has revealed that human IKKβ can adopt an open conformation that enables it to form oligomers, whereas mutagenesis studies have established that two of the surfaces that mediate oligomer formation are critical for the activation of IKKβ in cells [14]. It has therefore been proposed that IKKβ dimers transiently associate with one another through these interaction surfaces to promote *trans* autophosphorylation as part of their activation mechanism. Consistent with an essential role for autophosphorylation, we found that in IKKα-deficient MEFs the specific IKKβ inhibitor BI605906 prevented the IL-1- or TNF-stimulated conversion of IKKβ into the active di-phosphorylated species, i.e. phosphorylated at both Ser^177^ and Ser^181^ [8].

In the present study we report the unexpected finding that TAK1 and IKKβ phosphorylate different serine residues in the activation loop of IKKβ and demonstrate that the TAK1-catalysed phosphorylation of IKKβ at Ser^177^ is a priming event that enables IKKβ to activate itself by phosphorylating Ser^181^. We also provide genetic evidence showing that the formation of Met^1^-linked ubiquitin chains and their interaction with NEMO is needed for the TAK1-catalysed phosphorylation of Ser^176^ (IKKα) and Ser^177^ (IKKβ), and that TAK1 activity is not required for the formation of either Lys^63^-linked or Met^1^-linked ubiquitin chains.

## EXPERIMENTAL

### Materials

Murine IL-1α and TNF were purchased from Peprotech and mouse M-CSF (macrophage colony-stimulating factor) from R&D Systems. Pam_3_CSK4 was from Invivogen and LPS (lipopolysaccharide) O55:B5 was from Enzo Life Science. The monophosphorylated peptide KELDQGpSLCTSFVGTLQ and the diphosphorylated peptide KELDQGpSLCTpSFVGTLQ (where pS is phosphoserine), corresponding to amino acids 171–187 of IKKβ with phosphoserine at Ser^177^ only or at both Ser^177^ and Ser^181^ respectively, were synthesized by Pepceuticals. The IKKβ inhibitor BI605906 [8] was provided by Dr Natalia Shpiro (University of Dundee, Dundee, U.K.) and the TAK1 inhibitor NG25 by Dr Nathanael Gray (Harvard Medical School, Boston, MA, U.S.A.) [11], whereas the TAK1 inhibitor 5Z-7-oxozeaenol was purchased from BioAustralis Fine Chemicals.

### Protein expression and purification

The IKKβ (IKKβ[D166A]) was expressed as a GST fusion protein in HEK (human embryonic kidney)-293T suspension cells and, after cell lysis, was purified from the cell extracts by chromatography on glutathione–Sepharose. The GST-fusion protein was released from the glutathione–Sepharose by cleavage of the GST tag with PreScission protease. A catalytically active TAK1–TAB1 (TAK1-binding protein 1)-fusion protein [15] was expressed in insect Sf21 cells as a His_6_-tagged protein and purified by chromatography on nickel-nitrilotriacetate agarose. The catalytic subunit of human PP1γ (protein phosphatase 1γ) was expressed in *Escherichia coli* as a GST-fusion protein, purified on glutathione–Sepharose and stored in a solution of 50 mM Tris/HCl, 0.15 M NaCl, 0.27 M sucrose, 0.03% Brij35, 0.1% 2-mercaptoethanol and 2 mM MnCl_2_.

### Antibodies

An antibody recognizing the HOIP {HOIL1 [haem-oxidized IRP2 (iron regulatory protein 2) ubiquitin ligase 1]-interacting protein} component of LUBAC (linear ubiquitin chain assembly complex) was raised in sheep and purified as described in [16]. Antibodies recognizing IKKβ phosphorylated at Ser^177^ (catalogue number 2078S) or at both Ser^177^ and Ser^181^ (catalogue number 2697L) were obtained from Cell Signaling Technology, whereas the antibody recognizing IKKβ phosphorylated at Ser^181^ was from Abcam (catalogue number AB55341). Antibodies that recognize p105/NF-κB1 phosphorylated at Ser^933^ (catalogue number 4806S), GAPDH (glyceraldehyde-3-phosphate dehydrogenase; catalogue number 2118S) and all forms of p38α MAPK (catalogue number 9212S) and JNK (catalogue number 9258S) were from Cell Signaling Technology. Antibodies recognizing NEMO (catalogue number SC8330; Santa Cruz Biotechnology), all forms of IKKβ (catalogue number DAM1774677; Millipore) and the HA (haemaglutinnin) tag (catalogue number 12-013-819-001; Roche) were from the sources indicated. An antibody raised in sheep against the full-length human IKKβ catalytic subunit (S189C, bleed 1) was produced and affinity purified by the Antibody Production Team of the MRC Protein Phosphorylation and Ubiquitylation Unit at Dundee (co-ordinated by Dr James Hastie). An antibody recognizing Met^1^-linked ubiquitin chains was generously provided by Vishva Dixit, Genentech, U.S.A. and the antibody recognizing Lys^63^-linked ubiquitin chains was purchased from Merck-Millipore (catalogue number 05-1313).

### DNA constructs

DNA encoding IKKβ (NCBI BAI45894.1) was amplified from total thymus RNA using the One Step RT PCR kit (Life Technologies). It was then cloned into pCR2.1 (Life Technologies), sequenced and sub-cloned into the Not1 site of pRetrox tight HA. Mutations were created following the QuikChange Site-Directed Mutagenesis method, but using KOD Hot Start DNA Polymerase (EMD Millipore).

### Cell culture, stimulation and immunoblotting

MEFs and HEK-293 cells were maintained in DMEM (Dulbecco's modified Eagle's medium) supplemented with 2 mM glutamine, 10% (v/v) FBS, and the antibiotics streptomycin (0.1 mg/ml) and penicillin (100 units/ml). DNA constructs were transfected into HEK-293 cells using polyethyleneimine (Polysciences). BMDMs (bone-marrow-derived macrophages) were obtained by culturing bone marrow from the tibia and femurs of mice in the presence of mouse M-CSF and replating for 24 h before stimulation. Kinase inhibitors (10 mM) dissolved in DMSO, or an equivalent volume of DMSO for the control incubations, were added to the culture medium of cells grown as monolayers. After 1 h at 37°C, MEFs were stimulated with IL-1α or TNF and BMDM with LPS or Pam_3_Csk_4_ (see the Figure legends). Thereafter, cells were rinsed in ice-cold PBS and extracted in lysis buffer [50 mM Tris/HCl (pH 7.4), 1 mM EDTA, 1 mM EGTA, 50 mM NaF, 5 mM sodium pyrophosphate, 10 mM sodium 2-glycerol 1-phosphate, 1 mM DTT, 1 mM sodium orthovanadate, 0.27 M sucrose, 1% (v/v) Triton X-100, 1 μg/ml aprotinin, 1 μg/ml leupeptin and 1 mM PMSF]. Cell extracts were clarified by centrifugation (21000 ***g*** for 10 min at 4°C) and protein concentrations determined by the Bradford assay. Cell extract protein (20 μg) was separated by SDS/PAGE (8% gel), transferred on to PVDF membranes and proteins detected by immunoblotting and visualized by treating the blots with enhanced chemiluminescence (Amersham).

### Generation of MEFs from knockin mice

Mice in which wild-type NEMO was replaced by the polyubiquitin-binding-defective mutant NEMO[D311N] were generated by Taconic-Artemis using conventional technology and their characterization will be reported elsewhere. Primary MEFs from NEMO[D311N] mice and wild-type littermates were generated at E11.5 (embryonic day 11.5), whereas MEFs from knockin mice expressing the inactive C879S mutant of HOIP were generated at E10.5 [16]. Immortalized IKKα-deficient MEFs and wild-type control MEFs were provided by Dr Inder Verma (Salk Institute, La Jolla, CA, U.S.A.). All animals were maintained in specific pathogen-free conditions consistent with EU and U.K. regulations. All work was performed under a U.K. Home Office project license that was awarded after recommendation by the University of Dundee Ethical Review Committee.

### Retroviral transduction of IKKα-knockout MEFs

IKKα-deficient MEFs stably expressing HA-tagged empty vector (EV), wild-type HA–IKKβ (WT), HA–IKKβ[S177A], HA–IKKβ[S177E] or HA–IKKβ[D166A/S177E] were generated by retroviral transduction using an MMLV (Moloney murine leukaemia virus)-based system prepared with the VSVG (vesicular-stomatitis-virus glycoprotein) envelope protein. Retroviral particles were prepared according to the manufacturer’s instructions (Clontech). Viruses encoding the gene of interest and the Tet-On protein were harvested 48 h after transfection, diluted 4-fold with fresh medium and incubated for 24 h with IKKα-deficient MEFs in the presence of 2 μg/ml protamine sulfate (Sigma). Fresh medium containing 1 μg/ml G418 (Tet-On) and 3 μg/ml puromycin (gene of interest) was added to select the transduced cells. Cells were cultured for 16 h with doxycycline (0.1–1.0 μg/ml) to induce the expression of wild-type and mutant forms of IKKβ.

### Immunoprecipitation and dephosphorylation of IKKβ

To immunoprecipitate transfected HA-tagged IKKβ, cell extract protein (40 μg) was incubated for 60 min at 4°C with 4 μg of anti-HA antibody, whereas for the endogenous IKKβ 0.2 mg of cell extract protein was incubated with 2.5 μg of anti-IKKβ antibody. Protein G–Sepharose was added (equivalent to 10 μl packed volume) and, after mixing for 30 min at 4°C, immune complexes were collected by brief centrifugation, washed three times in cell lysis buffer plus 0.5 M NaCl, and three times with 50 mM Tris/HCl (pH 7.5), 0.05 M NaCl and 1.0 mM DTT, then resuspended in 0.03 ml of 50 mM Hepes, 10 mM NaCl, 2 mM DTT and 0.1% Brij35 (pH 7.5) containing 1 mM MnCl_2_. Dephosphorylation was initiated by the addition of 100 μg of GST–PP1γ. After 60 min at 30°C the immunoprecipitates were collected, washed three times with 1.0 ml of lysis buffer containing 0.5 M NaCl, and three times with 50 mM Tris/HCl (pH 7.5), 0.1 mM EGTA and 0.1% 2-mercaptoethanol to remove the phosphatase.

### Assay of immunoprecipitated IKKβ

IKKβ immunoprecipitates were assayed for IKKβ activity in a 0.05 ml incubation containing 50 mM Tris/HCl (pH 7.5), 0.1 mM EGTA and 0.1% 2-mercaptoethanol, 1.0 μM microcystin (to inactivate any remaining traces of PP1γ), 0.3 mM of the peptide LDDRHDSGLDSMKDEEY (corresponding to amino acid residues 26–42 of IκBα), 10 mM magnesium acetate and 0.1 mM [γ^32^P]ATP (5×10^5^ c.p.m./nmol). After incubation for 10 min at 30°C on a shaking platform, the incorporation of ^32^P radioactivity into the peptide substrate was measured as described in [17].

## RESULTS

### IKKβ is activated by TAK1 and by autophosphorylation

**Figure 1:**
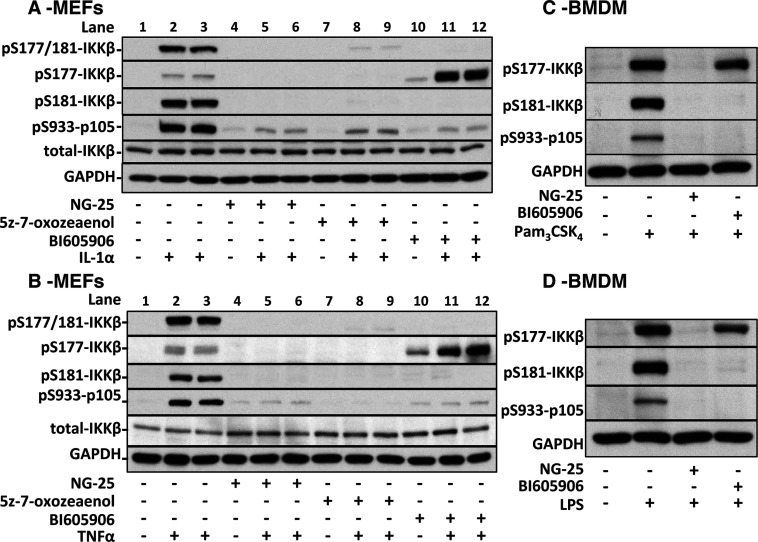
Effect of protein kinase inhibitors on the phosphorylation of IKKβ at Ser^177^ and/or Ser^181^ in MEFs from IKKα-deficient mice and BMDMs from knockin mice expressing catalytically inactive IKKα[S176A/S180A] (**A**) MEFs from IKKα-knockout mice were incubated for 1 h without (−) or with (+) 1.0 μM NG25, 1.0 μM 5Z-7-oxozeaenol or 5.0 μM BI605906, then stimulated for 10 min with 5.0 ng/ml IL-1. Cell lysates were subjected to SDS/PAGE and immunoblotting as described in the Experimental section. (**B**) The experiment was performed exactly as in (**A**) except that the cells were stimulated with 10 ng/ml TNF. (**C** and **D**) BMDMs from knockin mice expressing the catalytically inactive IKKα[S176A/S180A] mutant were incubated for 1 h without (−) or with (+) 2 μM NG25 or 2 μM BI605906, then stimulated for 10 min with 1 μg/ml Pam_3_CSK_4_ (**C**) or 0.1 μg/ml LPS (**D**). Cell extracts were subjected to SDS/PAGE and immunoblotted as in (**A** and **B**).

We initially confirmed that IL-1 or TNF stimulate the dual phosphorylation of IKKβ at Ser^177^ and Ser^181^ in IKKα-deficient MEFs, and that this was prevented by the inclusion of the IKKβ inhibitor BI605906 in the culture medium (Figures 1A and 1B, top panel, compare lanes 1–3 with 10–12). In these and many earlier studies, the phospho-specific antibody used to monitor the phosphorylation of IKKβ recognizes the di-phosphorylated species phosphorylated at both Ser^177^ and Ser^181^. It was therefore possible that BI605906 and/or pharmacological inhibitors of TAK1 had suppressed the phosphorylation of just one of the serine residues in the activation loop. To address this possibility we therefore employed antibodies that recognize IKKβ phosphorylated at either Ser^177^ or Ser^181^. These studies led to the striking and surprising observation that BI605906 suppressed the IL-1- or TNF-stimulated phosphorylation of Ser^181^, but not the phosphorylation of Ser^177^ (Figures 1A and 1B, second and third panels from top, lanes 10–12). In contrast, two structurally unrelated inhibitors of TAK1, NG25 and 5Z-7-oxozeaenol, prevented IL-1 or TNF from inducing the phosphorylation of IKKβ at both Ser^177^ and Ser^181^ in IKKα-deficient MEFs (Figures 1A and 1B, second and third lanes from top, lanes 4–9). Similar results were observed in BMDMs from knockin mice expressing the catalytically inactive IKKα[S176A/S180A] mutant (Figures 1C and 1D) [18].

The recognition of IKKβ by the Ser^177^ phospho-specific antibody appeared to be greatly enhanced when IKKα-deficient MEFs were incubated with BI605906 and then stimulated with IL-1 or TNF (Figures 1A and 1B, second panel from top, compare lanes 10–12 with 1–3). This observation is explained by the failure of the antibody to recognize IKKβ phosphorylated at Ser^177^ if Ser^181^ is also phosphorylated, and is not a reflection of a real increase in the phosphorylation of Ser^177^. This was shown by immunoblotting experiments with a synthetic mono-phosphorylated peptide corresponding to amino acid residues 171–187 of IKKβ containing phospho-serine at the position equivalent to Ser^177^, and a diphosphorylated form of this peptide with phosphoserine present at both Ser^177^ and Ser^181^ (Supplementary Figures S1A and S1B at http://www.biochemj.org/bj/461/bj4610531add.htm). We have encountered similar situations with other proteins in which the two sites of phosphorylation are separated by only four amino acid residues (e.g. [19]). In contrast, the antibody that recognizes the Ser^181^-phosphorylated form of IKKβ detected the di-phosphorylated form of the peptide (Supplementary Figure S1C), because this antibody recognizes the epitope Cys-Thr-pSer-Phe-Val (where pSer is phospho-Ser^181^), which does not contain Ser^177^. As expected, the antibody recognizing Ser^181^ of IKKβ did not detect the mono-phosphorylated peptide containing phospho-serine only at Ser^177^ (Supplementary Figure S1C).

**Figure 2:**
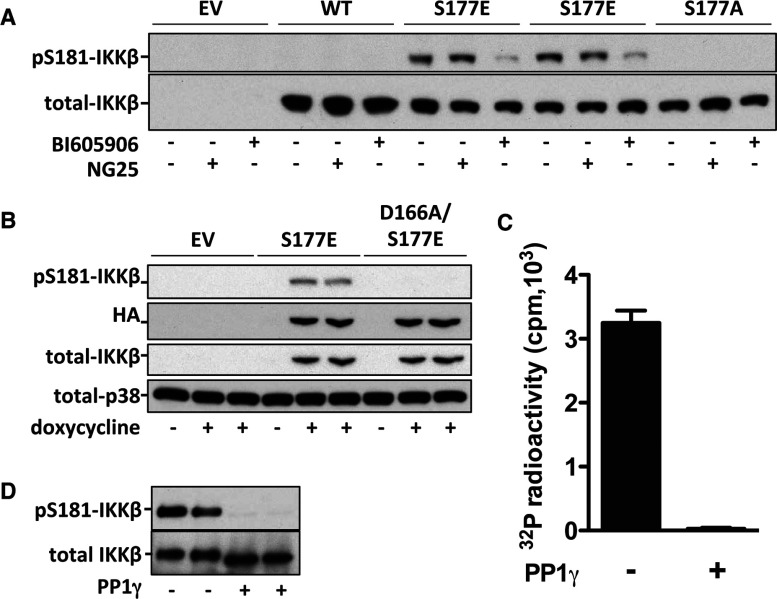
Expression of IKKβ[S177E] induces the autophosphorylation of Ser^181^ and activation of IKKβ (**A**) MEFs from IKKα-deficient mice stably expressing HA-tagged wild-type IKKβ (WT), IKKβ[S177E] (S177E) or IKKβ[S177A] (S177A) or empty vector (EV) were incubated for 16 h with 1.0 μg/ml (WT and EV), 0.2 μg/ml (S177A) or 0.1 μg/ml (S177E) doxycycline to induce the expression of these proteins, and then for 1 h without (−) or with (+) 5 μM NG25 or 5 μM BI605906 and lysed. Extract [20 μg (EV, S177E and S177A) or 80 μg (WT) protein] were analysed by immunoblotting with the antibodies indicated. (**B**) As in (**A**), except that Ser^181^ phosphorylation was studied in MEFs stably expressing HA–IKKβ[D166A/S177E] and HA–IKKβ[S177E], and no inhibitors were present. (**C** and **D**) HA-tagged IKKβ[S177E] was transfected into HEK-293 cells, immunoprecipitated from the cell extracts, incubated without (−) or with (+) PP1γ and assayed for IKKβ activity (**C**) or immunoblotted with antibodies that recognize IKKβ phosphorylated at Ser^181^ or all forms of IKKβ (**D**). The results in (**C**) are means±S.E.M. of duplicate determinations. Similar results were obtained in two other independent experiments.

The simplest interpretation of the results presented in Figure 1 was that the TAK1-catalysed phosphorylation of Ser^177^ was a prerequisite for the subsequent IKKβ-catalysed phosphorylation of Ser^181^. To investigate this hypothesis, we generated IKKα-deficient MEFs that stably expressed (under an inducible promoter) mutated forms of IKKβ in which Ser^177^ was changed to either glutamic acid (to mimic the effect of phosphorylation by introducing a negative charge) or to alanine (to prevent phosphorylation). The S177E mutant became phosphorylated at Ser^181^, even in MEFs that had not been stimulated with IL-1 or TNF, whereas the S177A mutant or wild-type IKKβ did not (Figure 2A). Moreover, under these conditions, incubation with the IKKβ inhibitor BI605906 induced substantial dephosphorylation of the S177E mutant at Ser^181^, whereas incubation with the TAK1 inhibitor NG25 had no effect. Furthermore, a catalytically inactive version of the S177E mutant, created by additionally mutating Asp^166^ in the Asp-Phe-Gly motif to alanine, failed to undergo phosphorylation at Ser^181^ (Figure 2B). Taken together, these experiments demonstrated, by two independent methods, that the phospho-mimetic S177E mutation permits IKKβ to autophosphorylate Ser^181^.

IKKβ initiates the activation of NF-κB *in vivo* by phosphorylating the inhibitory IκBα component at Ser^32^ and Ser^36^. The IKKβ[S177E]-catalysed phosphorylation of a synthetic peptide comprising amino acid residues 26–42 of IκBα was suppressed by BI605906 similarly to wild-type IKKβ (Supplementary Figures S2 at http://www.biochemj.org/bj/461/bj4610531add.htm), establishing that the activity being measured was catalysed by IKKβ and not by another protein kinase present in the immunoprecipitates as a contaminant. Phosphatase treatment inactivated the IKKβ[S177E] mutant (Figure 2C), and this was accompanied by the dephosphorylation of Ser^181^ and a small increase in the electrophoretic mobility of IKKβ (Figure 2D). These experiments established that the phospho-mimetic S177E mutation had not activated IKKβ, but permitted IKKβ to auto-activate by phosphorylating Ser^181^.

**Figure 3:**
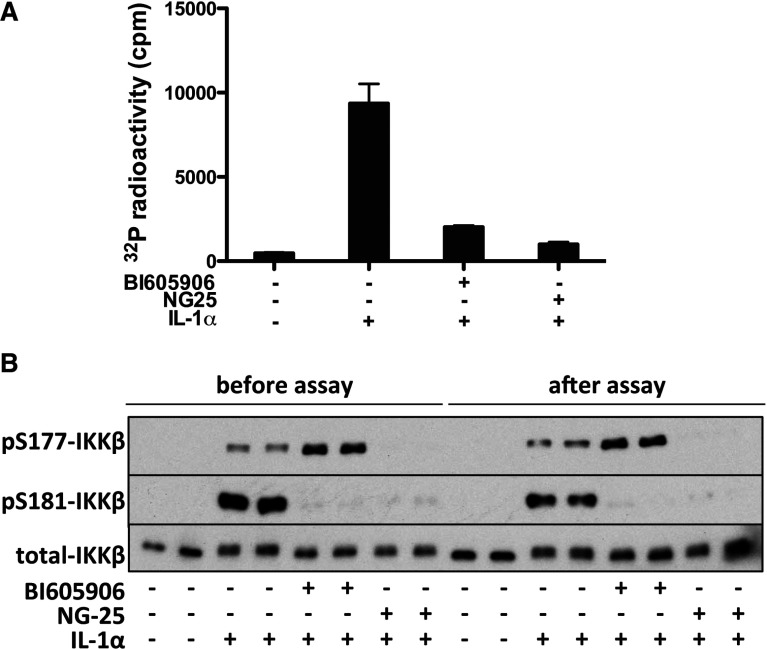
IKKβ phosphorylated at Ser^177^ has little activity if Ser^181^ is not phosphorylated (**A**) MEFs from IKKα-deficient mice were incubated for 1 h without (−) or with (+) 5.0 μM BI 605906 or 2 μM NG25, then stimulated for 10 min with 5.0 ng/ml IL-1. The endogenous IKKβ was immunoprecipitated from 0.2 mg of cell extract protein and assayed for activity. (**B**) The immunoprecipitates from (**A**) were denatured in SDS before and after the assay of IKKβ, and aliquots of each sample were subjected to SDS/PAGE, transferred on to PVDF membranes and immunoblotted with antibodies that recognize IKKβ phosphorylated at Ser^177^ or Ser^181^ or all forms of IKKβ.

BI605906 is a reversible inhibitor of IKKβ (Supplementary Figure S3 at http://www.biochemj.org/bj/461/bj4610531add.htm). To investigate whether the phosphorylation of Ser^177^ could activate IKKβ in the absence of Ser^181^ phosphorylation, we incubated IKKα-deficient MEFs with BI605906 to supress the phosphorylation of Ser^181^ and assayed the endogenous IKKβ activity after its immunoprecipitation from the extracts of IL-1-stimulated cells. These experiments showed that IKKβ mainly phosphorylated at Ser^177^ had a much lower activity than IKKβ phosphorylated at both Ser^177^ and Ser^181^ (Figure 3A). Taken together, the results presented in Figures 2 and 3 indicate that Ser^177^ is a priming event that enables IKKβ to auto-activate itself by phosphorylating Ser^181^.

### Activation of the canonical IKK complex

The experiments presented above were carried out in IKKα-deficient MEFs or in BMDMs from knockin mice expressing the catalytically inactive IKKα[S176A/S180A] mutant, because IKKα activity is unaffected by BI605906 [8]. In contrast, the phosphorylation of IKKβ at Ser^181^ was only decreased slightly by BI605906 in wild-type MEFs (Supplementary Figures S4A and S4B at http://www.biochemj.org/bj/461/bj4610531add.htm) and was not decreased significantly in Pam_3_CSK_4_- or LPS-stimulated BMDMs (Supplementary Figures S4C). This suggests that in wild-type cells, in which IKKα, IKKβ and NEMO form a single ternary complex, phosphorylation of IKKβ at Ser^181^ can be catalysed *in trans* by IKKα if IKKβ is inhibited by BI605906.

### The formation of Met^1^-linked ubiquitin chains and their interaction with NEMO is required for TAK1 to phosphorylate IKKα and IKKβ at Ser^176^/Ser^177^

**Figure 4:**
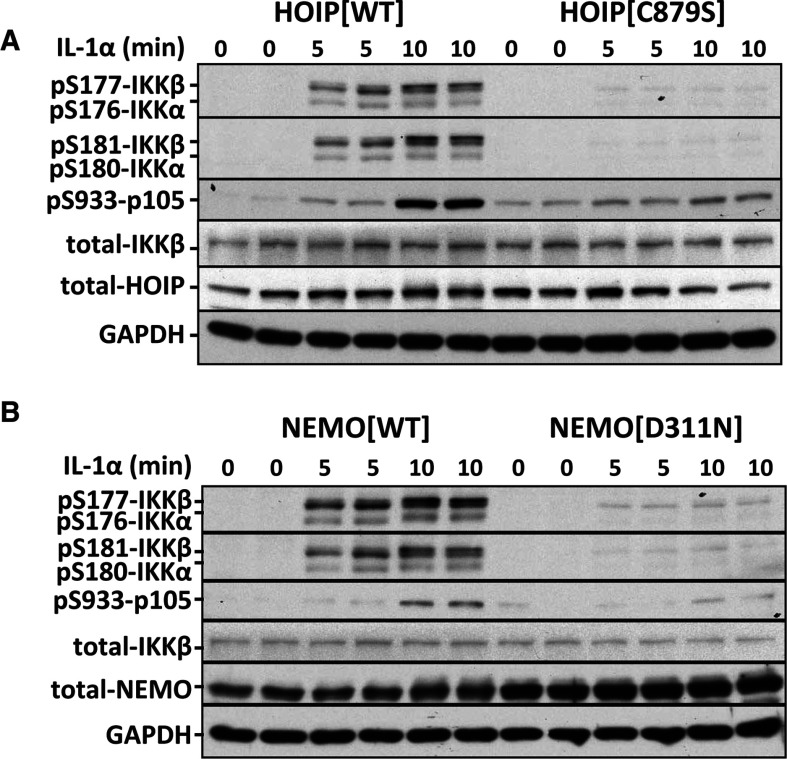
Met^1^-linked ubiquitin chains and their interaction with NEMO are required for the IL-1-stimulated phosphorylation of IKKα and IKKβ in MEFs (**A**) Cells from wild-type (HOIP[WT]) or knockin mice expressing the HOIP[C879S] mutant were stimulated with 5 ng/ml IL-1 for the times indicated and lysed. The extract (20 μg of protein) was subjected to immunoblotting and probed with the antibodies indicated. (**B**) As in (**A**) except that MEFs from NEMO[D311N]-knockin and wild-type mice were used.

LUBAC is the only E3 ubiquitin ligase that catalyses the formation of Met^1^-linked (linear) ubiquitin chains in IL-1-stimulated MEFs, and the formation of these ubiquitin chains is required for robust activation of the canonical IKK complex by this agonist ([16], reviewed in [3]). To investigate whether Met^1^-linked ubiquitin chain formation was required for the phosphorylation of Ser^177^, Ser^181^ or both amino acid residues, we studied the phosphorylation of each of these sites in MEFs from knockin mice in which HOIP, the catalytic subunit of LUBAC, was replaced by the inactive HOIP[C879S] mutant [16]. These experiments demonstrated that the IL-1-stimulated phosphorylation of IKKβ at Ser^177^ or Ser^181^ or IKKα at Ser^176^ or Ser^180^ was greatly reduced in MEFs from HOIP[C879S]-knockin mice, as was the phosphorylation of p105/NFκB1 at Ser^933^, an established physiological substrate of IKKβ (Figure 4A) [20].

The Met^1^-linked ubiquitin chains formed by LUBAC bind to the NEMO component of the canonical IKK complex (reviewed in [3]). We therefore generated knockin mice expressing NEMO[D311N], an ubiquitin-binding-defective mutant of NEMO [21–23], and studied the phosphorylation of IKKβ in MEFs from these animals. We found that the IL-1-stimulated phosphorylation of IKKβ at Ser^177^ or Ser^181^, or IKKα at Ser^176^ or Ser^180^, was impaired in MEFs expressing the NEMO[D311N] mutant (Figure 4B), similar to the results obtained in MEFs from the HOIP[C879S]-knockin mice (Figure 4A).

The IL-1-stimulated phosphorylation of JNK1/JNK2 and p38α MAPK in MEFs from HOIP[C879S] or NEMO[D311N] mice was similar to wild-type MEFs (Supplementary Figure S5 at http://www.biochemj.org/bj/461/bj4610531add.htm), but was suppressed by the TAK1 inhibitors NG25 or 5Z-7-oxozeaenol (Supplementary Figure S6 at http://www.biochemj.org/bj/461/bj4610531add.htm). These control experiments indicated that activation of the TAK1 complex was unimpaired in MEFs from HOIP[C879S]- or NEMO[D311N]-knockin mice. The TAK1 inhibitor NG25 did not affect the IL-1-stimulated formation of Lys^63^-linked ubiquitin chains significantly and actually enhanced Met^1^-linked ubiquitin chain production in IKKα-deficient MEFs (Supplementary Figure S7 at http://www.biochemj.org/bj/461/bj4610531add.htm). Thus TAK1 activity is not required for the IL-1-stimulated formation of Lys^63^-linked or Met^1^-linked ubiquitin chains and NG25 does not suppress the phosphorylation of IKKβ by preventing formation of the ubiquitin chains. The enhanced formation of Met^1^-linked ubiquitin chains in the presence of NG25 implies the existence of a TAK1-dependent feedback control mechanism for restricting the formation of these ubiquitin chains.

Finally, it should be noted that although TAK1 phosphorylates the IKKβ–NEMO complex at Ser^177^ in IKKα-deficient MEFs, the active TAK1 catalytic subunit is capable of phosphorylating a catalytically inactive mutant of the IKKβ catalytic subunit at Ser^181^, as well as Ser^177^, *in vitro* (Supplementary Figure S8 at http://www.biochemj.org/bj/461/bj4610531add.htm). It is therefore possible that the interaction of NEMO with IKKβ in the canonical IKK complex and/or the recruitment of the TAK1 complex to Lys^63^-linked ubiquitin chains are factors that prevent TAK1 from phosphorylating Ser^181^ in cells.

## DISCUSSION

**Figure 5:**
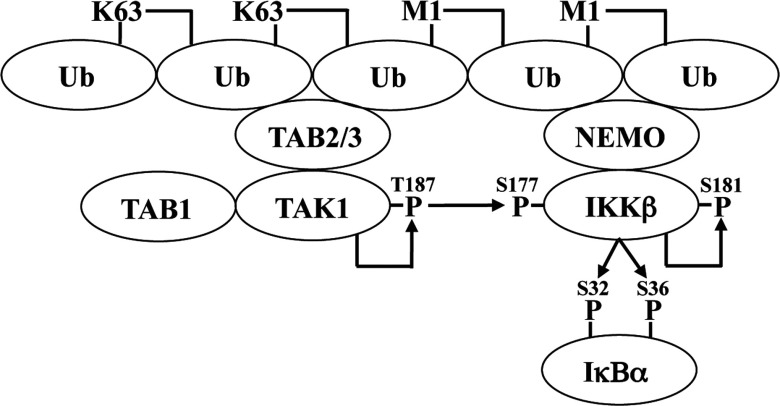
Proposed mechanism for the activation of IKKβ by IL-1 in IKKα-deficient MEFs IL-1 stimulates the formation of hybrid ubiquitin chains in which LUBAC-generated Met^1^-linked ubiquitin oligomers are attached covalently to TRAF6-generated Lys^63^-linked ubiquitin oligomers. The binding of Lys^63^-linked ubiquitin to TAB2 or TAB3 activates the TAK1 complex by inducing autophosphorylation of the catalytic subunit at Thr^187^ [31]. The M1-linked ubiquitin chains interact with NEMO permitting TAK1 to phosphorylate IKKβ at Ser^177^. Phosphorylation of Ser^177^ allows autophosphorylation of Ser^181^. The activated IKKβ can then phosphorylate IκBα. Sites of phosphorylation are denoted by ‘P’.

In the present study, we have clarified the mechanism by which the canonical IKK complex is activated. Unexpectedly, we discovered that the activation of IKKβ requires two sequential phosphorylation events. The activation process is initiated by the TAK1-catalysed phosphorylation of IKKβ at Ser^177^, which is a priming event that permits IKKβ to phosphorylate itself at Ser^181^, which is needed before IKKβ can phosphorylate exogenous substrates, such as IκBα (Figure 5). We have shown that this mechanism of activation operates in IL-1- or TNF-stimulated MEFs and in TLR-stimulated BMDMs indicating that is likely to be of general significance. However, the identity of the ‘priming’ kinase may vary from cell to cell.

The mutation of Ser^177^ of IKKβ to glutamic acid (to mimic the effect of phosphorylation by introducing a negative charge) permitted the IKKβ catalytic subunit to autophosphorylate at Ser^181^ and this induced activation even in cells that had not been stimulated with IL-1 or TNF. Interestingly, the other two members of the IKK subfamily of protein kinases, IKKε and TBK1 {TANK [TRAF (TNF receptor-associated factor)-associated NF-κB activator]-binding kinase 1}, both possess a glutamic acid at position 168 in their activation loops, which is the amino acid residue equivalent to Ser^176^/Ser^177^ of IKKα/β, and they are activated by the phosphorylation of Ser^172^, the site equivalent to Ser^180^/Ser^181^ of IKKα/IKKβ [8]. These features explain why these IKK-related kinases are not activated directly by TAK1 *in vivo* and why they are instead activated by the canonical IKK complex and by autophosphorylation in response to IL-1 [8]. Once activated, IKK-related kinases restrict the activity of the canonical IKKs by phosphorylating inhibitory sites on the canonical IKKs, which is critical to prevent autoimmune nephritis in mice [8,24].

The activation of the canonical IKK complex by IL-1 does not just require the phosphorylation of serine residues in the activation loop, but also the formation of a hybrid ubiquitin chain containing both Lys^63^-linked and Met^1^-linked ubiquitin oligomers [16]. The Lys^63^-linked ubiquitin chains interact with the TAB2 and TAB3 components of TAK1 complexes, in-ducing the auto-activation of TAK1 [9,13,25], whereas the Met^1^-linked ubiquitin chains formed by the action of the E3 ubiquitin ligase LUBAC [16,26] interact with NEMO [27,28] and are critical for activation of the canonical IKK complex [12,16,29]. Nearly all of the Met^1^-linked ubiquitin chains formed in response to IL-1 are attached covalently to Lys^63^-linked ubiquitin chains, which may facilitate the TAK1-dependent activation of canonical IKK complex by recruiting both protein kinases to the same ubiquitin chains [16]. In the present study, we found that the IL-1-stimulated phosphorylation of IKKα/IKKβ at Ser^176^/Ser^177^, and hence the phosphorylation of Ser^180^/Ser^181^, was suppressed in MEFs that were unable to produce Met^1^-linked ubiquitin chains or that expressed a ubiquitin-binding-defective mutant of NEMO (Figure 4). Thus the formation of Met^1^-linked ubiquitin chains and their interaction with NEMO are both needed for TAK1 to phosphorylate IKKα/IKKβ at Ser^176^/Ser^177^ and so enable the IKKs to complete the activation process by phosphorylating Ser^180^/Ser^181^ (Figure 5).

The activation of many protein kinases requires the phosphorylation of two amino acid residues within their activation loops. For example, similar to the canonical IKK complex, the seven members of the MKK family undergo dual phosphorylation at Ser/Thr-Xaa-Xaa-Xaa-Ser/Thr (where Xaa is any amino acid residue) sequences, enabling them to activate their cognate MAPKs. Similarly, most MAPKs are activated by the dual phosphorylation of a threonine and a tyrosine residue that are located in Thr-Xaa-Tyr within their activation loops. Although the activation of many MKKs and MAPKs is thought to be catalysed by a single protein kinase, the present study has shown that the requirement for one ‘upstream’ protein kinase does not exclude the possibility that a second protein kinase is also required. Indeed, we have shown that the activation of JNK requires the MKK7-catalysed phosphorylation of the threonine and the MKK4-catalysed phosphorylation of the tyrosine residue within the Thr-Xaa-Tyr motif [30]. The activation of a kinase by two different ‘upstream’ kinases provides additional opportunities for signal integration if each activating kinase responds to distinct physiological cues. We suggest that this situation may be a more frequent occurrence than has hitherto been realized, and that this is a neglected area that merits further attention.

## Online data

Supplementary data
