# Supplementary material for: An unexpected twist to the activation of IKKβ: TAK1 primes IKKβ for activation by autophosphorylation
Source: Biochem J. 2014 Jul 10;461(Pt 3):531–7. doi: 10.1042/BJ20140444 (PMC4206954; doi:10.1042/BJ20140444)
Supplement: Supplementary data [file bj4610531add.pdf]

BJ Signal

Biochem. J. (2014) , 531–537 (Printed in Great Britain) doi:10.1042/BJ20140444

online data

SUPPLEMENTARY ONLINE DATA

An unexpected twist to the activation of IKKβ: TAK1 primes IKKβ for activation by autophosphorylation

Jiazhen ZHANG*, Kristopher CLARK*, Toby LAWRENCE†, Mark PEGGIE* and Philip COHEN*[1]

*MRC Protein Phosphorylation and Ubiquitylation Unit, College of Life Sciences, University of Dundee, Dundee DD1 5EH, Scotland, U.K.
†Centre d'Immunologie de Marseille-Luminy, Parc Scientifique & Technologique de Luminy, Case 906, 13288 Marseille cedex 09, France

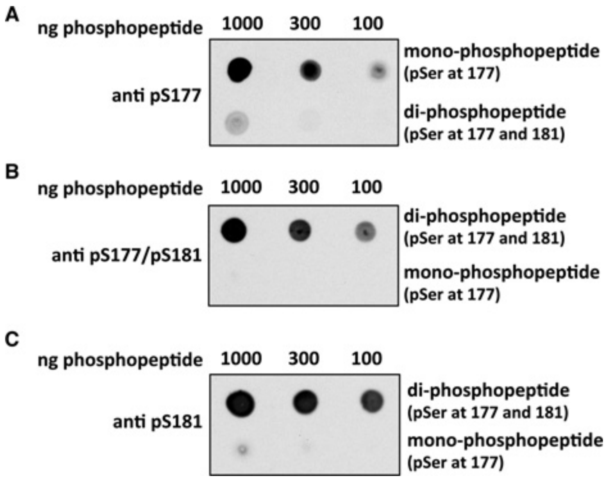

Figure S1 Phosphorylation of Ser[181] interferes with the recognition of phosphor-Ser[177] by the phospho-specific antibody that recognizes this site

(**A**) The indicated amounts of phosphopeptides corresponding to amino acid residues 171–187 of IKKβ phosphorylated at Ser[177] only or at both Ser[177] and Ser[181] were spotted on to nitrocellulose membranes and probed with the phospho-specific antibody recognizing phospho(p)-Ser[177]. (**B**) Same as (**A**), except that the peptides were immunoblotted with the antibody that only recognizes the di-phosphorylated form of IKKβ phosphorylated at both Ser[177] and Ser[181]. (**C**) Same as (**A**), except that the phosphopeptides were immunoblotted with the antibody that recognizes IKKβ phosphorylated at Ser[181].

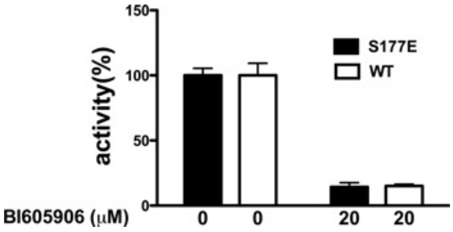

Figure S2 Effect of BI605906 on the activity of wild-type and mutant IKKβ

HA-tagged wild-type IKKβ (WT) or the IKKβ[S177E] mutant (S177E) were expressed in HEK-293 cells, immunoprecipitated from the cell extracts using an anti-HA antibody and assayed for activity in the absence or presence of BI605906. The activities are plotted as a percentage of that obtained in the absence of inhibitor. Results are means±S.E.M. of duplicate determinations. Similar results were obtained in another independent experiment.

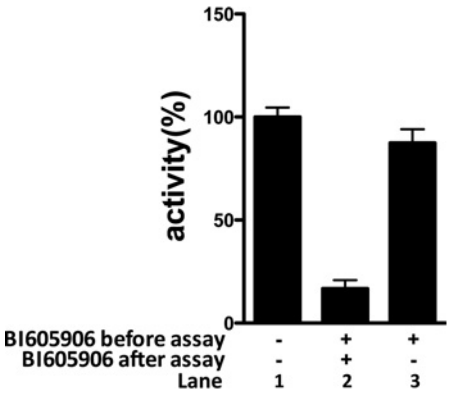

Figure S3 BI605906 is a reversible inhibitor of IKKβ

MEFs from IKKα-deficient mice were stimulated for 10 min with 5.0 ng/ml IL-1 and the cells were lysed. The endogenous IKKβ was immunoprecipitated from 0.2 mg of cell extract protein and incubated for 1 h at 30°C without (−, lane 1) or with (+, lanes 2 and 3) 20 μM BI605906. In lane 3 only, the immunoprecipitates were washed extensively to remove BI605906. All the immunoprecipitates were then assayed for IKKβ activity. The Figure shows that IKKβ activity was full restored after washing away the inhibitor. Results are means ± S.E.M. of duplicate determinations. Similar results were obtained in another independent experiment.

1 To whom correspondence should be addressed (email p.cohen@dundee.ac.uk).

© The Authors Journal compilation © 2014 Biochemical Society

Biochemical Journal www.biochemj.org

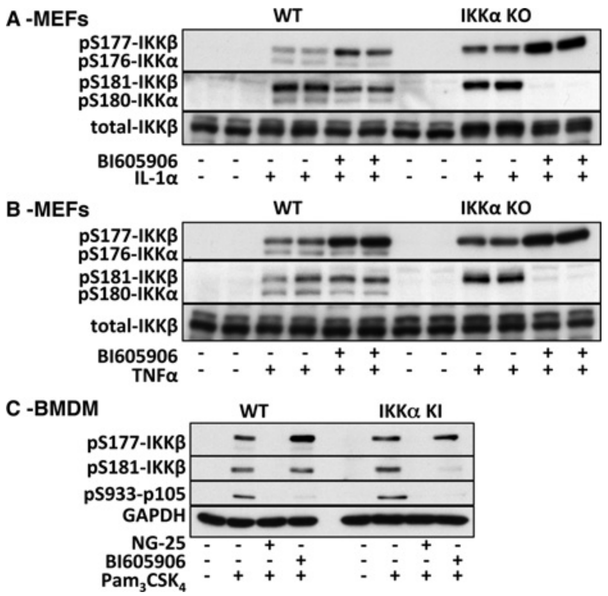

Figure S4 Effect of inhibitors on agonist-stimulated phosphorylation of IKKβ at Ser[177] and Ser[181] in MEFs and BMDM

(**A** and **B**) MEFs from wild-type (WT) or IKKα-knockout (KO) mice were incubated for 1 h without ($-$) or with ($+$) 5.0 μM BI 605906 and then stimulated for 10 min with 5.0 ng/ml IL-1 (**A**) or 10 ng/ml TNF (**B**). Following cell lysis, cell extract (20 μg of protein) was denatured in SDS, subjected to SDS/PAGE, and immunoblotted with antibodies that recognize IKKα and IKKβ phosphorylated at Ser[176] and Ser[177] respectively, or with antibodies that recognize IKKα and IKKβ phosphorylated at Ser[180] or Ser[181] respectively. The membranes were also immunoblotted with antibodies that recognize all forms of IKKβ. (**C**) Same as (**A** and **B**), except that BMDMs from wild-type (WT) mice and knockin (KI) mice expressing the catalytically inactive mutant of IKKα were used and the cells were stimulated for 10 min with 1.0 μg/ml Pam_3CSK_4. An antibody recognizing GAPDH (glyceraldehyde-3-phosphate dehydrogenase) was used as a loading control.

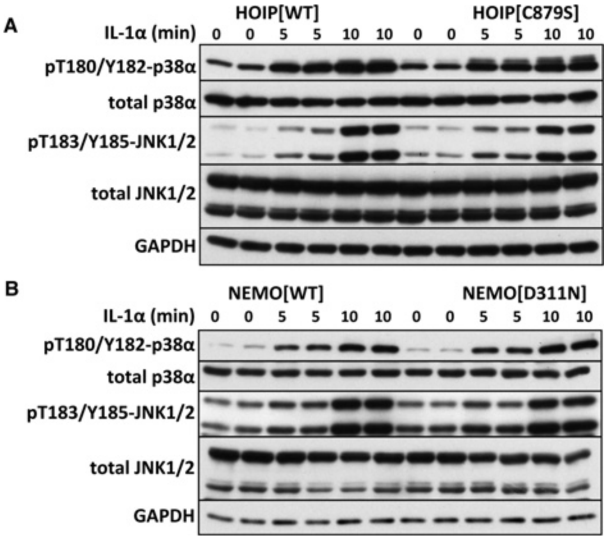

Figure S5 Phosphorylation of MAPKs is unimpaired in MEFs from HOIP[C879S] and NEMO[D311N] mice

(**A**) MEFs from wild-type mice (HOIP[WT]) or knockin mice expressing the inactive HOIP[C879S] mutant were stimulated with 5.0 ng/ml IL-1 for the times indicated. After cell lysis, 20 μg of cell extract protein was denatured in SDS, subjected to SDS/PAGE and immunoblotted with the antibodies indicated. (**B**) Same as (**A**), but using MEFs from mice expressing the polyubiquitin-binding-deficient mutant of NEMO (NEMO[D311N]). The antibody recognizing pT183/Y185 of JNK1/2 was from Invitrogen and the antibody recognizing pT180/Y182 of p38α MAPK was from Cell Signaling Technology.

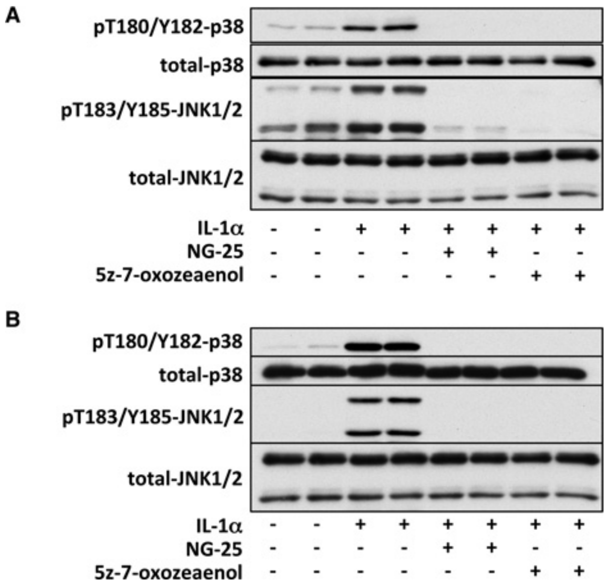

Figure S6 TAK1 inhibitors suppress the IL-1-stimulated phosphorylation of MAPKs in MEFs from HOIP[C879S]- and NEMO[D311N]-knockin mice

(**A**) As in Figure S5, except that before stimulation with IL-1, MEFs from mice expressing the HOIP[C879S] mutant were incubated for 1 h without ($-$) or with ($+$) the TAK1 inhibitors NG25 (1.0 μM) or 5Z-7-oxozeaenol (1.0 μM) before stimulation with 5.0 ng/ml IL-1 for the times indicated. (**B**) Same as (**A**), except that the experiment was performed with MEFs from mice expressing the polyubiquitin-binding-deficient mutant NEMO[D311N].

© The Authors Journal compilation © 2014 Biochemical Society

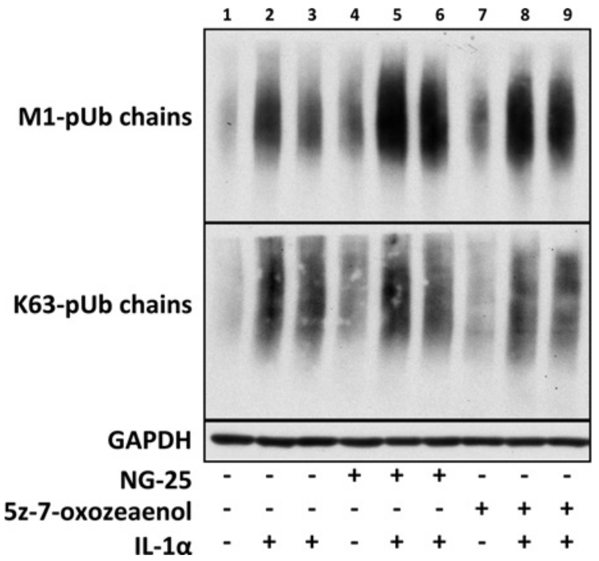

Figure S7 Effect of TAK1 inhibition on the IL-1-stimulated formation of Lys^{63}-linked and Met^{1}-linked ubiquitin chains

MEFs were incubated for 1 h with (+) or without (−) 2 μM NG25 or 1 μM 5Z-7-oxozeaenol, then stimulated for 10 min with 5 ng/ml IL-1α and lysed. The Met^{1}-linked and Lys^{63}-linked ubiquitin chains present in 2 mg of cell extract protein were captured on Halo-NEMO [1], released by denaturation in SDS and immunoblotted with antibodies that recognize Met^{1}-linked or Lys^{63}-linked ubiquitin chains specifically. The same cell extracts (20 μg of protein) were immunoblotted with an anti-GAPDH antibody as a loading control.

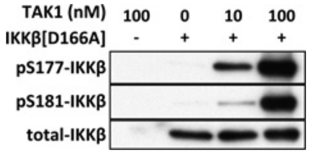

Figure S8 TAK1 phosphorylates IKKβ at Ser^{177} and Ser^{181} *in vitro*

Catalytically inactive IKKβ[D166A] (0.8 μM) was incubated for 3 min at 30 °C with the indicated concentrations of the active TAK1–TAB1 fusion protein in 50 mM Tris/HCl (pH 7.5), 0.1 mM EGTA, 2 mM DTT, 10 mM magnesium acetate and 0.1 mM ATP. Reactions were terminated by denaturation in SDS and, after SDS/PAGE and transfer on to PVDF membranes, proteins were immunoblotted with antibodies that recognize IKKβ phosphorylated at Ser^{177} or Ser^{181} or antibodies recognizing all forms of IKKβ.

REFERENCES

1 Emmerich, C. H., Ordureau, A., Strickson, S., Arthur, J. S., Pedrioli, P. G., Komander, D. and Cohen, P. (2013) Activation of the canonical IKK complex by K63/M1-linked hybrid ubiquitin chains. Proc. Natl. Acad. Sci. U.S.A. **110**, 15247–15252 CrossRef PubMed

Received 4 April 2014/20 May 2014; accepted 9 June 2014
Published as BJ Immediate Publication 9 June 2014, doi:10.1042/BJ20140444

© The Authors Journal compilation © 2014 Biochemical Society
